# Characterization and optimization of a zein-based mesoscopic complex system for *Venenum Bufonis* delivery

**DOI:** 10.3389/fnut.2026.1916662

**Published:** 2026-10-20

**Authors:** Peizhi Mao, Shiyin Chen, Fuge Niu, Zhaie Lu, Weichun Pan

**Affiliations:** 1 Women and Children's Hospital of Ningbo University, Ningbo, China; 2 College of Food Science and Biotechnology, Zhejiang Gongshang University, Hangzhou, China

**Keywords:** drug delivery system, encapsulation, gastrointestinal digestion, mesoscopic complex, physicochemical properties, *Venenum Bufonis* (VB), zein

## Abstract

*Venenum Bufonis* (*VB*), a traditional Chinese medicine with multiple pharmacological activities, exhibits promising therapeutic potential but is limited by poor aqueous solubility, low bioavailability, and gastrointestinal irritation caused by its toxic components. Developing an effective delivery system is therefore critical for improving its application potential. In this study, zein was employed as a biocompatible carrier to encapsulate *VB* and construct zein-based mesoscopic complexes (MCS). Four zein-to-*VB* mass ratios were investigated, and the physicochemical properties, encapsulation performance, stability, and *in vitro* gastrointestinal release behaviors of the obtained complexes were systematically evaluated. Fourier transform infrared spectroscopy (FTIR) analysis suggested that hydrogen bonding and hydrophobic interactions contributed to the association between zein and *VB*. Among the investigated formulations, the zein–*VB* complex prepared at a zein-to-*VB* mass ratio of 10:1 exhibited the most favorable overall performance, with the highest encapsulation efficiency (98.29%) and moderate drug loading capacity (9.04%). Furthermore, this formulation demonstrated improved storage stability and sustained *VB* release behavior, with a cumulative release rate of 49.0% under simulated gastrointestinal conditions. The zein-based mesoscopic complex system effectively improved *VB* dispersibility and provided controlled release characteristics, which may reduce gastrointestinal irritation and enhance *VB* utilization efficiency. This study provides a potential strategy for developing plant protein-based delivery systems for *VB* and other poorly water-soluble bioactive compounds.

## 1 Introduction

*Venenum Bufonis* (*VB*) is prepared by drying the white serous secretion obtained from the postauricular and skin glands of *Bufo bufo gargarizans* or *Duttaphrynus melanostictus*. As a traditional Chinese medicine, *VB* has documented diverse pharmacological activities, including cardiovascular regulation, analgesic effects, anti-inflammatory activity, and antitumor potential (1). The major bioactive constituents of *VB*, including cinobufagin, bufalin, and resibufogenin, are bufadienolide compounds with notable anticancer activities through multiple mechanisms, such as the induction of tumor cell apoptosis, inhibition of angiogenesis, and modulation of immune responses (2). For instance, bufalin induces apoptosis in human colorectal cancer HCT116 cells by activating the endoplasmic reticulum stress pathway (3). Cinobufacini has been reported to alleviate lung tumor progression and suppress epithelial–mesenchymal transition (EMT) activity (4). Furthermore, resibufogenin inhibits the proliferation, migration, invasion, and EMT of multiple myeloma cells by blocking the PI3K/AKT signaling pathway (5).

Despite its promising pharmacological effects, the clinical application of *VB* remains restricted by several limitations. First, the major active components of *VB* are highly lipophilic bufadienolides, resulting in poor aqueous solubility and limited bioavailability. Second, *VB* exhibits considerable irritation toward blood vessels and the gastrointestinal tract. Moreover, its rapid elimination and extensive tissue distribution contribute to a relatively short biological half-life, requiring frequent administration and potentially reducing patient compliance. In addition, the cardiotoxicity associated with *VB* further narrows its therapeutic window. Therefore, the development of an efficient delivery strategy is urgently needed to overcome the challenges of toxicity, poor solubility, low bioavailability, and dose-related safety concerns (1).

An efficient drug delivery system capable of improving *VB* solubility, enhancing bioavailability, and enabling controlled release may provide a promising approach to address these limitations. Previous studies have demonstrated that nanocarrier-based systems can improve the therapeutic performance of *VB*-related compounds. For example, solid lipid nanoparticles increased the oral bioavailability of bufalin by 4.2-fold (6). Liposomal encapsulation of butotryptamine reduced intestinal exposure and significantly alleviated gastrointestinal irritation (7). In addition, various nanostructured carriers, including Prussian blue nanocomplex and metal–organic framework-based systems, have been developed to achieve targeted delivery and reduce systemic toxicity (8, 9).

Among various nanocarrier materials, plant proteins have attracted increasing attention due to their excellent biocompatibility, biodegradability, availability, and relatively low cost. Zein, a major corn-derived storage protein and a by-product of corn starch processing, possesses unique self-assembly properties and high hydrophobicity. Its amino acid composition is enriched with proline, leucine, alanine, and phenylalanine, allowing effective encapsulation of hydrophobic bioactive compounds (10). Moreover, zein has been recognized as Generally Recognized As Safe (GRAS) by the US Food and Drug Administration (FDA), supporting its broad applications in food, pharmaceutical, and biomedical industries (11).

Several approaches have been developed to prepare zein-based delivery systems, including antisolvent precipitation, emulsification-solvent evaporation, pH-driven assembly, and self-assembly methods. Among these strategies, the antisolvent precipitation method has gained considerable attention due to its simplicity, mild conditions, and suitability for encapsulating hydrophobic compounds (12). Zein-based nanocarriers generally exhibit favorable characteristics, including biocompatibility, biodegradability, enhanced solubility of hydrophobic compounds, and controlled release capability (13). Previous studies have demonstrated that zein-based delivery systems can improve the stability and bioavailability of various functional compounds, such as curcumin, quercetin, vitamin D3, and ω-3 polyunsaturated fatty acids (14–17).

Although zein-based delivery systems have been extensively investigated for functional compounds, the encapsulation of *VB* using zein-based mesoscopic complexes has not yet been reported. Therefore, this study aimed to construct and characterize zein–*VB* mesoscopic complexes (MCS) with different zein-to-*VB* ratios. The effects of formulation composition on particle characteristics, encapsulation efficiency, stability, and *VB* release behavior under simulated gastrointestinal conditions were systematically investigated. The findings may provide a potential strategy for improving *VB* delivery efficiency while reducing its gastrointestinal irritation and toxicity, thereby promoting its future pharmaceutical applications.

## 2 Materials and methods

### 2.1 Materials

Zein was purchased from Shandong Keyuan Biochemical Co., Ltd. (Heze, China). *Venenum Bufonis* (*VB*) was generously provided by Jiangsu Jingchan Pharmaceutical Co., Ltd. (Huaian, China) and was obtained from the dried secretion of the Chinese giant toad (*Bufo bufo gargarizans*). Ethanol, potassium dihydrogen phosphate (KH_2_PO_4_), hydrochloric acid (HCl, ca12 mol L^−1^), and sodium hydroxide (NaOH) were of analytical grade and purchased from Xilong Chemical Co., Ltd. (Shantou, China).

Pepsin (≥500 U/mg) and trypsin (8 × USP) were obtained from Sigma-Aldrich (Shanghai, China). Porcine bile salt was purchased from Shanghai Macklin Biochemical Technology Co., Ltd. (Shanghai, China). All reagents were used as received without further purification. Ultrapure water was produced using a Millipore-Q device (Merck, Burlington, MA).

### 2.2 Zein–*VB* mesoscopic complex system

Zein–*VB* mesoscopic complexes (MCS) were prepared according to a previously reported antisolvent precipitation method with minor modifications (17).

Briefly, zein (0.04 g) and different amounts of *VB* were dissolved in 20 mL of 60% (v/v) ethanol aqueous solution to obtain zein-to-*VB* mass ratios of 5:1, 10:1, 20:1, and 30:1. After complete mixing, the solutions were treated using an ultrasonic shaker (F-020SD, Shenzhen Fuyang Technology Group Co., Ltd., Shenzhen, China) at 35 ± 1 °C for 10 min.

Subsequently, the mixtures were stirred at 4,000 rpm for 2 h and then freeze-dried using a lyophilizer (SCIENTZ-10N, Ningbo Scientz Freeze Dryer Equipment Co., Ltd., Ningbo, China) to obtain zein–*VB* complex powders.

The obtained complexes were designated as Zein–*VB*_5:1_, Zein–*VB*_10:1_, Zein–*VB*_20:1_, and Zein–*VB*_30:1_ according to their zein-to-*VB* mass ratios. All samples were stored in sealed bags at 4 °C and analyzed within 48 h.

### 2.3 Zeta potential assessment

The zeta potential of zein–*VB* MCS was measured using a Zetasizer Nano ZS90 instrument (Malvern Instruments, Malvern, UK) at room temperature (approximately 25 °C).

Prior to measurement, samples were diluted to a zein concentration of 1 mg mL^−1^ and equilibrated for 120 s. Each sample was measured three times independently, with 30 scans performed per measurement.

### 2.4 Scanning electron microscopy (SEM)

The morphology of zein–*VB* MCS was examined using field-emission scanning electron microscopy (FE-SEM; Sigma 300, Zeiss, Oberkochen, Germany) following a previously reported procedure with minor modifications (18).

Briefly, a small amount of the freeze-dried sample was directly mounted onto conductive adhesive tape and coated with gold for 45 s using a Quorum SC7620 sputter coater at 10 mA under vacuum conditions. SEM observations were performed at an accelerating voltage of 3.0 kV.

### 2.5 Encapsulation efficiency and drug loading capacity assessment

The encapsulation efficiency (*EE*) and loading capacity (*LC*) of zein–*VB* MCS were determined using a high-speed centrifugation method.

Briefly, 1 mL of the sample suspension was centrifuged at 10,000 × g for 20 min at 10 °C using a TGL-16M centrifuge (Hunan Xiangyi Laboratory Equipment Development Co., Ltd., Changsha, China). The supernatant was collected, and the concentration of free *VB* was determined by high-performance liquid chromatography (HPLC).

The *EE* and *LC* values were calculated according to Equations 1, 2:


$$\begin{array}{l} EE=\frac{C_{total}-C_{free}}{C_{total}}\times 100\% \end{array}$$ (1)

$$\begin{array}{l} LC=\frac{C_{total}-C_{free}}{W}\times 100\% \end{array}$$ (2)

where *C*_*total*_ represents the total *VB* concentration used during preparation, *C*_*free*_ represents the concentration of unencapsulated *VB* in the supernatant, and *W* represents the total mass of zein.

### 2.6 High-performance liquid chromatography analysis

The *VB* concentration was determined by HPLC according to a previously reported method with minor modifications (19).

An Agilent 1260 Infinity II HPLC system (Santa Clara, CA, USA) equipped with a C18 column (SunFire, 5 μm, 250 mm × 7.8 mm; Waters, Milford, MA, USA) was employed.

The mobile phase consisted of acetonitrile and 0.5% potassium dihydrogen phosphate solution (52:48, v/v), adjusted to pH 3.0. The detection wavelength was set at 296 nm. The flow rate was 0.500 mL min^−1^, the column temperature was maintained at 40 °C, and the injection volume was 10 μL.

### 2.7 Fourier transform infrared spectroscopy (FTIR)

FTIR spectroscopy was conducted to investigate the interactions between zein and *VB*.

Samples were mixed with potassium bromide (KBr) at a mass ratio of 1:100 and ground into fine powders before being compressed into pellets.

Spectra were recorded using a Nicolet iS5 spectrometer (Thermo Fisher Scientific, Waltham, MA, USA) at 25 °C over a wavenumber range of 4,000–400 cm^−1^ with a resolution of 4 cm^−1^. Each spectrum was collected from 32 scans, and measurements were performed at least three times for each sample.

#### *2.8 In vitro* gastrointestinal digestion and *VB* release

The *in vitro* digestion procedure was performed according to previously reported methods (20, 21) with minor modifications.

#### 2.8.1 Preparation of simulated gastric fluid (SGF)

SGF was prepared by diluting 0.5 mL of concentrated HCl with ultrapure water to 25 mL, followed by the addition of 1 g pepsin and 0.20 g NaCl. The final volume was adjusted to 100 mL, and the solution was ultrasonicated for 10 min at 35 °C.

#### 2.8.2 Preparation of simulated intestinal fluid (SIF)

SIF was prepared by dissolving 0.68 g NaH_2_PO4 and 1.00 g trypsin in ultrapure water to a final volume of 100 mL. After ultrasonication under the same conditions as SGF preparation, the solution was centrifuged at 10,000 × g for 10 min at 10 °C. Subsequently, 3.00 g porcine bile salts were added.

For digestion experiments, zein–*VB* MCS suspensions were mixed with SGF at a volume ratio of 1:1. The pH was adjusted to 2.0 using 1 mol L^−1^ HCl or NaOH solutions. The samples were incubated in a shaking water bath (SHA-B, Hangzhou Bonakeji Co., Ltd., Hangzhou, China) at 37 °C and 100 rpm.

Samples were collected at 0, 0.5, 1, and 1.5 h during gastric digestion. Subsequently, the digested samples were mixed with SIF at a volume ratio of 1:1, and the pH was adjusted to 6.8. The samples were collected at 0, 0.5, 1, and 1.5 h for *VB* release analysis.

The cumulative *VB* release was calculated according to Equation 3:


$$\begin{array}{l} Cumulative\;release=\frac{C\times V}{M}\times 100\% \end{array}$$ (3)

where *C*_*t*_ denotes the *VB* concentration released at time *t, V* represents the solution volume, and *M* represents the total amount of encapsulated *VB*.

### 2.9 Particle size measurement

Particle size and polydispersity index (PDI) were determined using an ALV/CGS-3 goniometer system (ALV, Langen, Germany) equipped with a He–Ne laser (λ = 632.8 nm).

Measurements were performed at 25 °C with a detection angle of 90° and a measurement duration of 30 s.

Via the intensity correlation function (Equation 4)


$$g_{2}(t)-1={\left(A1e^{-t/\tau_{1}}+A_{2}e^{-t/\tau_{2}}\right)}^{2}+\varepsilon$$ (4)

where τ_1_ and τ_2_ are the characterization time of particles in solutions; *A*_1_ and *A*_2_ are the corresponding amplitudes; and ε is the noise function (22). τ_*i*_ could be estimated by the software package offered by the company. Then, *R*_*H*_ could be determined via the Stokes–Einstein relation (Equation 5)


$$\begin{array}{l} R_{H,i}=\frac{k_{B}Tq^{2}}{6\pi \eta }\tau_{i} \end{array}$$ (5)

where *k*_*B*_ is the Boltzmann constant, *T* is the temperature, *q* is the wave-vector ($=\frac{4\pi nsin\left(\frac{\theta }{2}\right)}{\lambda }$, *n* is the refractive index of solvent; θ is the angle of the detector, =90°; λ is the wavelength of laser, 632.8 nm), and η is the viscosity of solution.

Via the cumulants method (Equation 6)


$$\begin{array}{l} \ln\;g_{1}\left(t\right)=\ln A-\bar{\Gamma }t+\frac{\mu_{2}}{2!}t^{2}-\frac{\mu_{3}}{3!}t^{3}+\cdots \end{array}$$ (6)

where $g_{1}\left(t\right)=A_{1}e^{-t/\tau_{1}}+A_{2}e^{-t/\tau_{2}}$; *A* is the intercept of the correlation function; $\bar{\Gamma }$ is the average decay rate; $PDI=\;\frac{\mu_{2}}{\bar{\Gamma }}$.

To evaluate stability, zein–*VB* MCS samples prepared at different zein-to-*VB* ratios (5:1, 10:1, 20:1, and 30:1) were stored in aqueous dispersion at room temperature for 0, 5, and 12 days.

The effects of thermal treatment and pH were also investigated. The samples were heated at 60, 80, or 100 °C for 1 h before particle size analysis. In addition, particle size measurements were conducted after dispersion in solutions with pH values of 2.0 and 12.0.

### 2.10 Statistical analysis

All experiments were performed at least in triplicate, and the results were expressed as mean ± standard deviation (SD).

Data analysis and visualization were performed using Origin 2024 software (OriginLab, Northampton, MA). Statistical significance was evaluated using a one-way analysis of variance (ANOVA), followed by Duncan's multiple range test using SPSS version 20.0 (IBM, USA).

## 3 Results

### 3.1 Characterization of zein–*VB*_5:1_ MCS

The hydrodynamic radius (*R*_*H*_) of zein–*VB*_5:1_ was 2,567 ± 55 nm, with a corresponding polydispersity index (PDI) of 1.01 ± 0.02 (Figure 1a). The relatively large particle size and broad size distribution indicate that the obtained structures were mesoscopic complexes rather than conventional nanoparticles. The particle size was considerably larger than those commonly reported for zein-based nanoparticles (approximately 100 nm) (23, 24).

**Figure 1:**
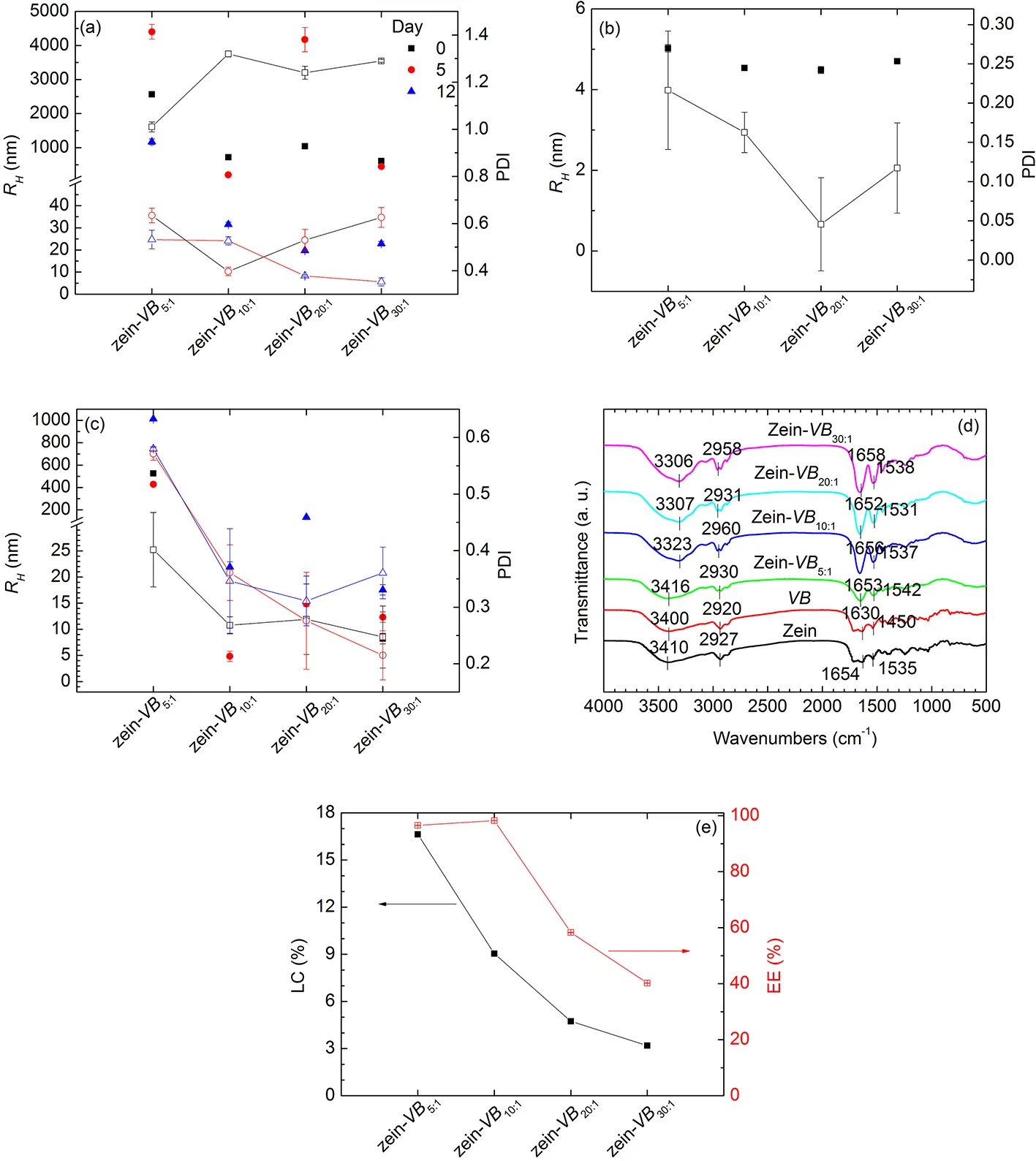
The characterization of zein–*VB* MCS (mean ± S.D.; *n* = 3), hydrodynamic radius and its PDI **(a)**, pH **(b)**, zeta-potential **(c)**, FTIR spectra **(d)**, and encapsulation efficiency and drug loading capacity (**e)**. Some error bars are so small that they are hard to be seen.

The pH value of the zein–*VB*_5:1_ suspension was 5.60 ± 0.01 (Figure 1b). The zeta potential was measured as 1.900 ± 0.390 mV (Figure 1c). Previous studies have demonstrated that zein nanoparticles generally exhibit positive surface charges under acidic conditions (25). The relatively low zeta potential observed in this system suggests that *VB* molecules may interact with zein surfaces and partially shield charged residues, thereby reducing the surface charge density.

FTIR analysis was performed to investigate the molecular interactions between zein and *VB* (Figure 1d). For zein, the broad absorption band around 3,410 cm^−1^ was attributed to O–H stretching vibrations, while the peaks at 2,927, 1,654, and 1,535 cm^−1^ corresponded to C–H stretching vibrations, amide I (C=O stretching), and amide II (N–H bending and C–N stretching), respectively. The characteristic α-helix-associated peak appeared at approximately 1,630 cm^−1^ (26).

For *VB*, characteristic peaks associated with O–H stretching, C–H stretching, ester carbonyl (C=O) stretching, C–H bending, and aromatic ring vibrations were observed at approximately 3,400, 2,920, 1,710, 1,450, and 830 cm^−1^, respectively (27).

Compared to pure zein and *VB*, zein–*VB*_5:1_ exhibited shifts in the characteristic peaks near 3,400 and 2,920 cm^−1^, indicating interactions between zein and *VB* molecules. These spectral changes suggest that hydrogen bonding and hydrophobic interactions may contribute to the formation of the zein–*VB* complexes.

The encapsulation efficiency *(EE*) of zein–*VB*_5:1_ reached 96.54 ± 0.06%, while the loading capacity (*LC*) was 16.63 ± 0.14% (Figure 1e). The high EE indicates a strong association between *VB* and zein, whereas the relatively high *LC* reflects sufficient *VB* incorporation at this formulation ratio.

SEM observations showed that zein–*VB*_5:1_ consisted of irregularly distributed particles with an average diameter of approximately 560 nm (Figure 2a and Supplementary Figure S1). The particle size distribution observed by SEM was consistent with the high PDI value obtained from light scattering measurements. Surface wrinkles and rough structures were observed, suggesting the possible localization of *VB* molecules near the particle surface. In addition, particle aggregation was observed, which may explain why the hydrodynamic size measured by light scattering was larger than the particle size observed by SEM.

**Figure 2:**
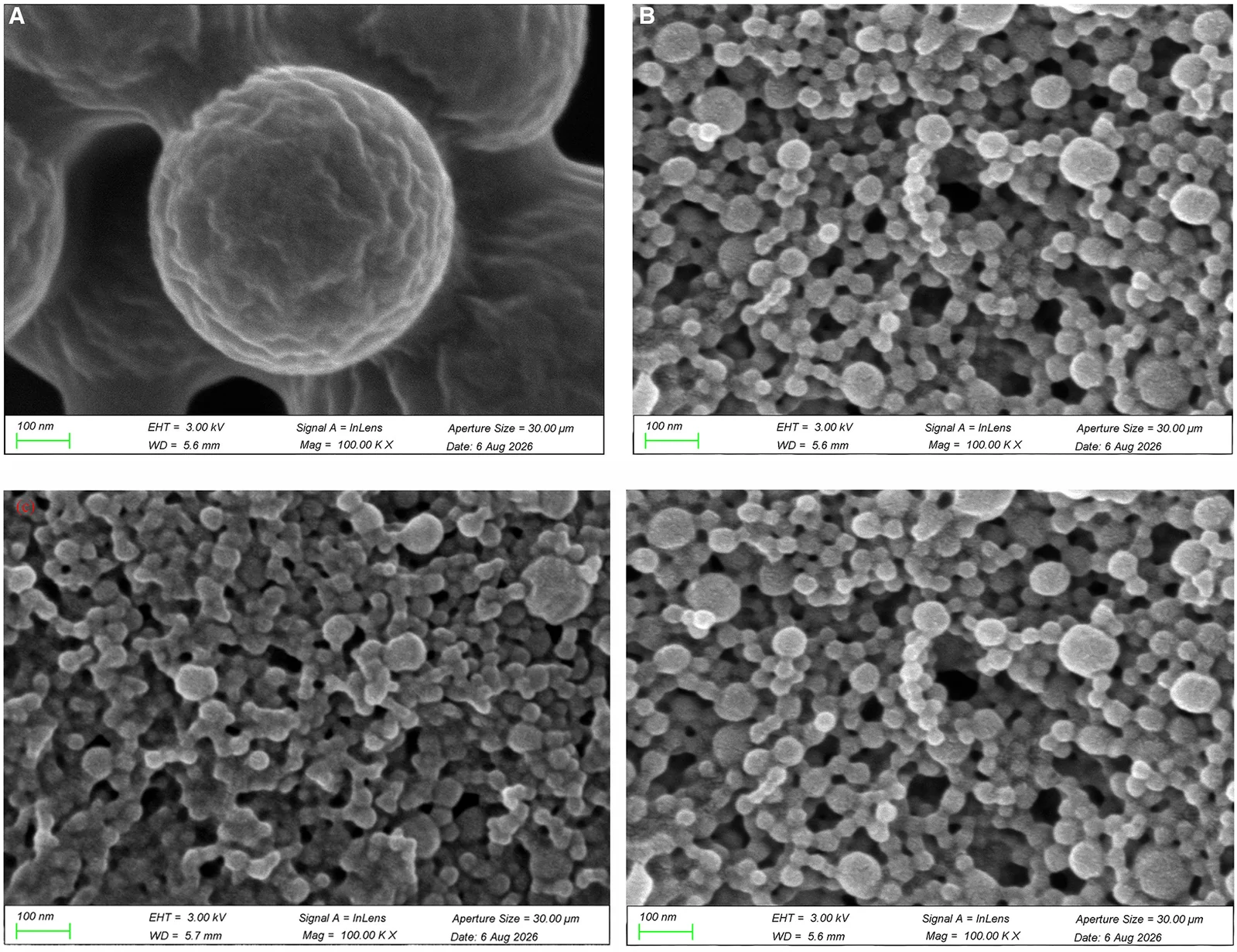
SEM images of zein–*VB*_5:1_ **(A)**; zein–*VB*_10:1_ **(B)**; zein–*VB*_20:1_ **(C)**; and zein–*VB*_30:1_ **(D)**.

The storage stability of zein–*VB*_5:1_ was further evaluated. During storage from day 0 to day 5, the *R*_*H*_ increased from 2,567 ± 55 nm to 4,403 ± 215 nm, followed by a decrease to 1,173 nm on day 12. Meanwhile, the PDI decreased continuously from 1.01 ± 0.02 to 0.53 ± 0.04 (Figure 3a). These results suggest that the MCS initially underwent aggregation, followed by partial structural rearrangement or precipitation of larger aggregates during prolonged storage.

**Figure 3:**
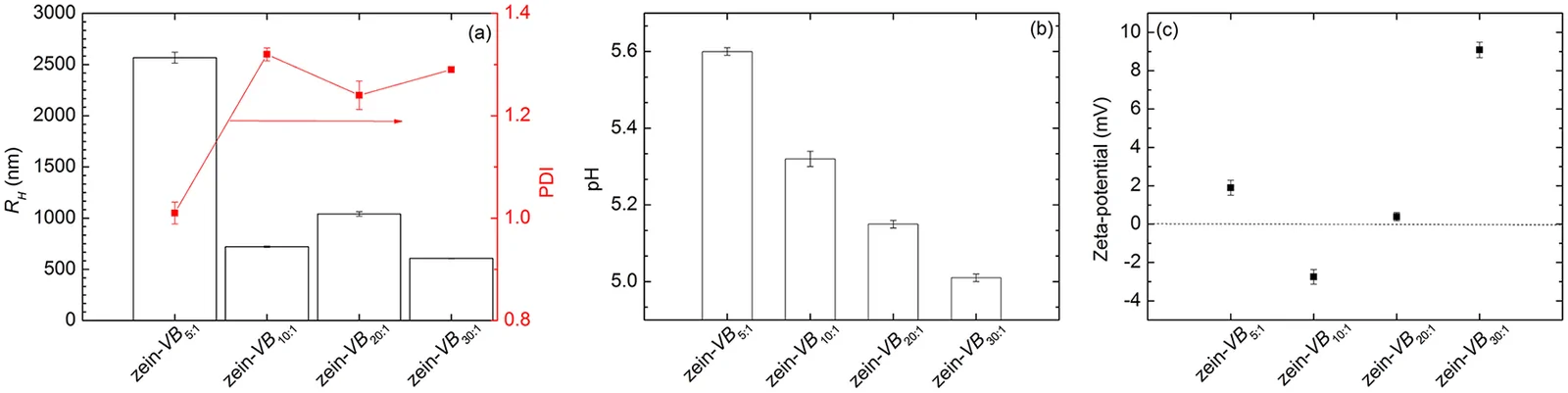
The stability of zein–*VB* MCS characterized by the storage period **(a)**, pH **(b)**, and temperature (mean ± S.D.; *n* = 3) **(c)**. Some are so small that they are hard to be seen. Black stands for 60 °C, red for 80 °C, and blue for 100 °C. Solid denotes for *R*_*H*_ while empty for PDI. In **(b)**, results of pH 2.0 did not presented since no detectable MCS existed.

The release behavior of *VB* from zein–*VB*_5:1_ was investigated under simulated gastrointestinal conditions (Figure 4). *VB* exhibited gradual release in both simulated gastric fluid (SGF) and simulated intestinal fluid (SIF). The release profile could be fitted by a first-order kinetic model, whereas other models including Higuchi, Hixson–Crowell, Korsmeyer–Peppas, and Weibull models showed poorer fitting performance (Supplementary Figure S6). The sustained release behavior may be related to the interaction between *VB* molecules and the internal structure of the zein complexes.

**Figure 4:**
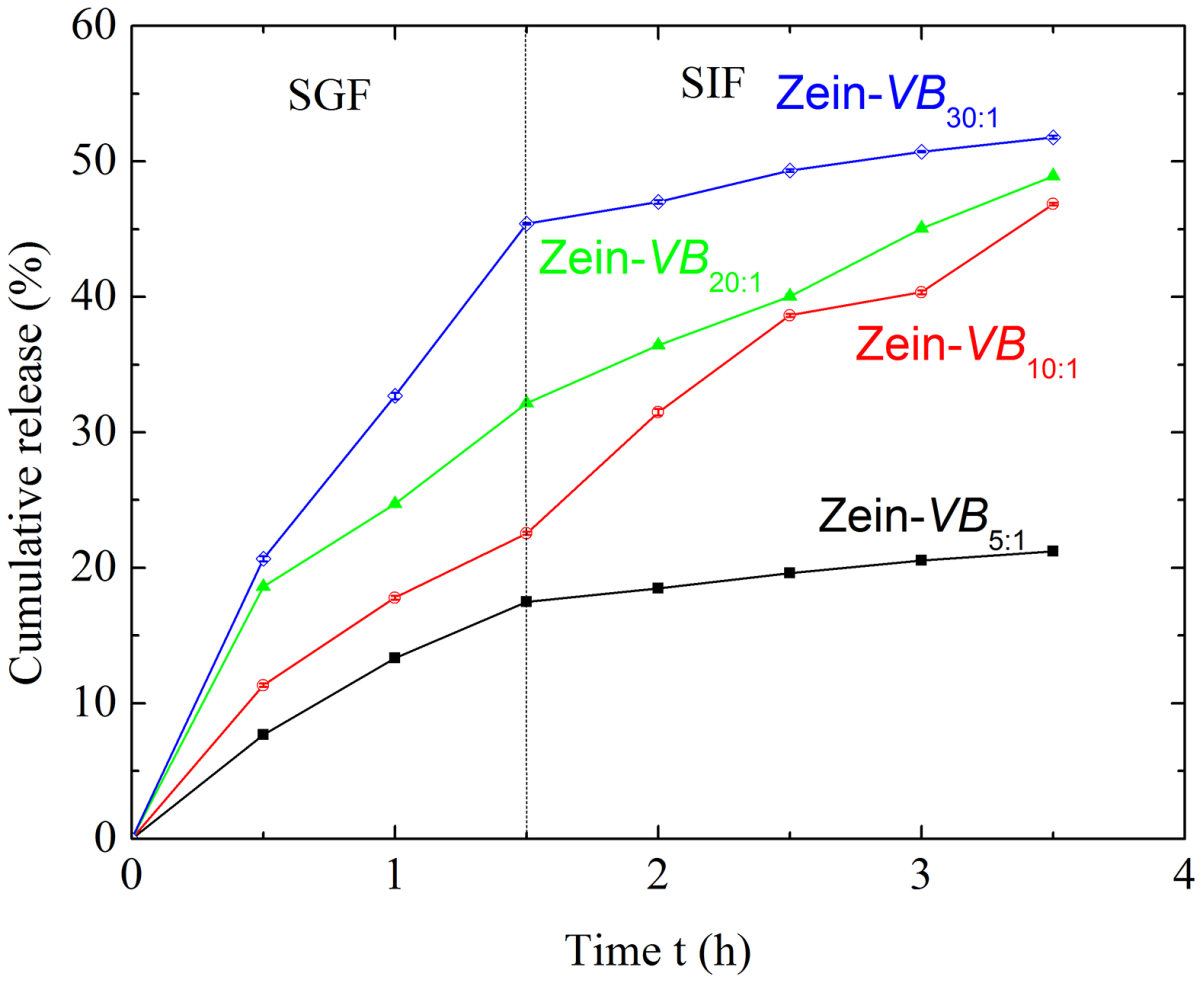
*In vitro* release profile of *VB* from zein–*VB* MCS in SGF and SIF. Lines of different colors stand for various MCS composition. Blue: zein–*VB*_30:1_; green: zein–*VB*_20:1_; red: zein–*VB*_10:1_; and black: zein–*VB*_5:1_.

### 3.2 Characterization of zein–*VB*_10:1_ MCS

The *R*_*H*_ of zein–*VB*_10:1_ was 721 ± 7 nm, with a PDI value of 1.32 ± 0.01 (Figure 1a). Compared to zein–*VB*_5:1_, the reduced particle size suggests that increasing the zein proportion influenced the assembly behavior of the complexes.

The suspension exhibited a pH value of 5.32 ± 0.02 (Figure 1b). Interestingly, the zeta potential was −2.747 ± 0.379 mV (Figure 1c), differing from the generally positive charge reported for zein nanoparticles. This phenomenon may be associated with conformational rearrangement of zein molecules after *VB* binding, exposing negatively charged residues while masking some positively charged regions.

FTIR analysis further supported structural changes caused by zein–*VB* interactions. Compared with other formulations, zein–*VB*_10:1_ exhibited more pronounced shifts near 3,400 and 2,920 cm^−1^. In addition, a shift near 1,656 cm^−1^ corresponding to α-helix-related structures was observed, suggesting that *VB* binding may induce changes in zein conformation.

Zein–*VB*_10:1_ exhibited the highest *EE* among all formulations, reaching 98.29 ± 0.06%, while the *LC* was 9.04 ± 0.09% (Figure 1e). Although the increased zein content reduced the relative *VB* proportion, it improved *VB* encapsulation efficiency.

SEM images revealed smaller and more uniform structures compared with zein–*VB*_5:1_ (Figure 2b and Supplementary Figure S2). The observed nanoparticles ranged from approximately 40–107 nm. The disappearance of surface wrinkles observed in zein–*VB*_5:1_ suggests that increasing zein content may alter the distribution of *VB* within the complex structure.

During storage, the *R*_*H*_ decreased from 721 ± 7 nm at day 0 to 199 ± 9 nm at day 5 and further decreased to 32 ± 1 nm at day 12 (Figure 3a). The continuous reduction in particle size indicates progressive structural dissociation or rearrangement of the complexes.

The *VB* release rate of zein–*VB*_10:1_ was slightly faster than that of zein–*VB*_5:1_ under simulated gastrointestinal conditions (Figure 4), possibly due to weaker *VB*–zein interactions or differences in particle structure.

### 3.3 Characterization of zein–*VB*_20:1_ MCS

The *R*_*H*_ of zein–*VB*_20:1_ was 1,041 ± 23 nm, with a PDI value of 1.24 ± 0.03 (Figure 1a). The pH value and zeta potential were 5.15 ± 0.01 and 0.384 ± 0.219 mV, respectively (Figures 1b, c).

The relatively low surface charge suggests altered residue distribution caused by zein–*VB* interactions. FTIR spectra showed a shift near 2,931 cm^−1^ (Figure 1d), indicating weaker molecular conformational changes compared to zein–*VB*_10:1_.

The *EE* and *LC* of zein–*VB*_20:1_ decreased substantially to 58.29 ± 0.11% and 4.74 ± 0.09%, respectively (Figure 1e). The excessive zein content may promote zein self-assembly through hydrophobic interactions, reducing available binding sites for *VB* molecules.

SEM images showed aggregated nanoparticles ranging from approximately 20–107 nm (Figure 2c and Supplementary Figure S3). Compared to zein–*VB*_10:1_, fewer large aggregates were observed.

During storage, the *R*_*H*_ initially increased from 1,041 ± 23 nm to 4,173 ± 357 nm and subsequently decreased to 20 ± 0 nm by day 12 (Figure 3a), indicating initial aggregation followed by structural disruption or precipitation.

Thermal stability analysis showed that zein–*VB*_20:1_ exhibited lower stability than zein–*VB*_5:1_ but higher stability than zein–*VB*_10:1_. Increasing temperature induced changes in particle size, suggesting complex temperature-dependent interactions between zein molecules and *VB* components.

The release of *VB* from zein–*VB*_20:1_ was faster than that from the previous formulations (Figure 4), which may be attributed to weaker interactions between *VB* and zein caused by excessive zein content.

### 3.4 Characterizations of zein–*VB*_30:1_ MCS

The *R*_*H*_ of zein–*VB*_30:1_ was 607 ± 3 nm, with a PDI of 1.29 ± 0.00 (Figure 1a). The suspension pH was 5.01 ± 0.01, and the zeta potential increased to 9.087 ± 0.405 mV (Figures 1b, c).

FTIR spectra showed peak shifts near 2,958 and 1,658 cm^−1^, suggesting changes in zein molecular conformation. However, the encapsulation performance decreased significantly, with *EE* and *LC* values of 40.27 ± 0.07% and 3.19 ± 0.09%, respectively (Figure 1e).

SEM analysis demonstrated nanoparticle assemblies ranging from approximately 20 to 220 nm (Figure 2c and Supplementary Figure S3). The particles exhibited more spherical morphologies compared with zein–*VB*_20:1_.

During storage, the *R*_*H*_ decreased continuously from 607 ± 3 nm to 445 ± 30 nm and finally to 23 ± 1 nm after 12 days (Figure 3a), indicating progressive structural rearrangement.

The influence of pH was dramatic. No detectable MCS existed when pH 2.0. But pH shifted to 12.0, small MCS observed at all zein contents with almost the same size in terms of RH and PDI (Figure 3b).

Among all formulations, zein–*VB*_30:1_ exhibited relatively high thermal stability. Particle sizes remained within the nanoscale range after heating at 60, 80, and 100 °C (Figure 3c).

However, *VB* release from zein–*VB*_30:1_ was the fastest among the four formulations (Figure 4), indicating weaker retention of *VB* within the complex structure.

## 4 Discussion

### 4.1 Potential reduction of *VB*-associated toxicity

The toxicity and irritation associated with *VB* remain the major limitations for its further application. In this study, the concentration of *VB* in the zein-based MCS system was maintained below approximately 400 μg mL^−1^, which is considerably lower than the concentration reported for intravenous administration (6 mg mL^−1^) (1).

The encapsulation of *VB* within zein-based complexes may reduce the direct exposure of free *VB* molecules to biological interfaces, thereby potentially decreasing gastrointestinal irritation and systemic toxicity. Furthermore, the controlled release behavior observed in simulated gastrointestinal conditions suggests that zein MCS may provide a sustained *VB* delivery profile rather than rapid exposure to high local concentrations.

However, further studies involving *in vivo* pharmacokinetic evaluation and toxicity assessment are required to confirm the safety advantages of this delivery system.

### 4.2 Influence of zein content on complex formation and *VB* encapsulation

The zein-to-*VB* mass ratio significantly influenced the physicochemical properties and encapsulation performance of the obtained MCS.

Zein molecules possess strong self-assembly capability, mainly driven by hydrophobic interactions and intermolecular disulfide bonding (28). When excessive zein was present, the self-assembly of zein molecules may compete with *VB* binding, resulting in fewer available hydrophobic regions for interaction with *VB* components. This phenomenon explains the reduced encapsulation efficiency and loading capacity observed in zein–*VB*_20:1_ and zein–*VB*_30:1_.

*VB* is a complex mixture containing various bufadienolide compounds with different polarities. Hydrophobic components may associate with the hydrophobic domains of zein, while hydroxyl and carbonyl groups may participate in hydrogen bonding interactions with zein functional groups. Therefore, variations in zein content may alter the balance between zein self-assembly and *VB*–zein interactions.

The particle size exhibited a non-linear relationship with zein content. The *R*_*H*_ values of zein–*VB*_5:1_, zein–*VB*_10:1_, zein–*VB*_20:1_, and zein–*VB*_30:1_ were 2,567, 721, 1,041, and 607 nm, respectively. The smallest particle size was obtained at zein–*VB*_30:1_, whereas the highest encapsulation efficiency was achieved at zein–*VB*_10:1_.

These results indicate that particle size alone does not determine encapsulation performance. Instead, the final properties of the MCS are governed by the complex interactions between zein self-assembly, *VB* binding, and particle structural organization.

SEM observations further revealed morphology differences among formulations. At a low zein content (zein–*VB*_5:1_), surface wrinkles and structural irregularities were observed, suggesting heterogeneous distribution of *VB* within the complexes. Increasing zein content reduced these structural features, indicating enhanced structural compactness.

Together, these results demonstrate that an appropriate zein-to-*VB* ratio is critical for achieving a balance between encapsulation efficiency, structural stability, and release behavior.

### 4.3 Stability of zein–VB MCS

The stability of zein-based delivery systems is essential for their practical application. In this study, storage stability, pH stability, and thermal stability were systematically evaluated.

Based on storage behavior, the formulations could be classified into two groups. Zein–*VB*_5:1_ and zein–*VB*_20:1_ showed an initial increase followed by a decrease in particle size during storage, whereas zein–*VB*_10:1_ and zein–*VB*_30:1_ exhibited continuous size reduction.

For zein–*VB*_5:1_ and zein–*VB*_20:1_, the relatively low surface charge may weaken electrostatic repulsion between particles, promoting aggregation during early storage. The subsequent decrease in particle size may result from precipitation of large aggregates or structural rearrangement toward more stable assemblies.

In contrast, the continuous size reduction observed in zein–*VB*_10:1_ and zein–*VB*_30:1_ may indicate gradual dissociation or reorganization of the mesoscopic complexes into smaller structures.

The surrounding pH environment also affected the stability of zein–*VB* MCS. At pH 2.0, few detectable complexes remained in suspension (Figure 1b), which may be attributed to protonation of surface residues and disruption of *VB*–zein interactions. At pH 12.0, smaller particles were observed, suggesting enhanced hydration and charge effects under alkaline conditions.

Temperature treatment further demonstrated the complex thermal responsiveness of zein–*VB* MCS. Heating initially caused particle disruption, followed by possible reassembly at higher temperatures. This behavior may originate from temperature-induced conformational changes of zein molecules and rearrangement of intermolecular interactions.

Compared with previously reported zein-based delivery systems, the behavior of zein–*VB* MCS appears to be strongly influenced by the complex composition of *VB*, indicating that the interaction mechanism differs from systems containing single purified bioactive compounds.

### 4.4 *In vitro* gastrointestinal release behavior

The release profiles of *VB* from zein MCS varied depending on formulation composition.

Except for zein–*VB*_10:1_, the release profiles of the other formulations could be described using a first-order kinetic model, although different fitting efficiencies were observed. Zein–*VB*_5:1_ showed the best agreement with the first-order model, followed by zein–*VB*_30:1_ and zein–*VB*_30:1_ (Supplementary Figure S6).

The sustained release behavior is likely related to the interaction between *VB* components and the zein matrix. Stronger hydrophobic interactions and hydrogen bonding may restrict *VB* diffusion, whereas weaker interactions may facilitate faster release.

Among the four formulations, zein–*VB*_10:1_ achieved a desirable balance between encapsulation efficiency and release behavior. Its high *EE* (98.29%) indicates effective *VB* incorporation, while its moderate release profile suggests potential for controlled delivery.

These findings suggest that optimization of the zein-to-*VB* ratio is crucial for regulating *VB* retention and release behavior.

## 5 Conclusion

In this study, zein-based mesoscopic complexes were successfully developed for *VB* encapsulation. The influence of zein-to-*VB* mass ratio on particle characteristics, surface charge, encapsulation efficiency, stability, and *in vitro* gastrointestinal release behavior was systematically investigated.

The obtained zein–*VB* complexes showed improved aqueous dispersibility compared with free *VB*, indicating that zein can serve as an effective carrier for this poorly water-soluble bioactive compound. Among the four formulations, zein–*VB*_10:1_ exhibited the most favorable overall properties, including the highest encapsulation efficiency (98.29%), moderate loading capacity (9.04%), improved storage stability, and controlled *VB* release behavior.

The zein-based MCS system may provide a promising strategy for improving *VB* delivery efficiency and reducing potential gastrointestinal irritation caused by rapid exposure to free *VB*. Further *in vivo* studies are required to validate its pharmacokinetic performance, biological efficacy, and safety profile.

## Data Availability

The raw data supporting the conclusions of this article will be made available by the authors, without undue reservation.
