# Supplementary material for: Characterization and optimization of a zein-based mesoscopic complex system for Venenum Bufonis delivery
Source: Front Nutr. 2026 Oct 20;13:1916662. doi: 10.3389/fnut.2026.1916662 (PMC13729855; doi:10.3389/fnut.2026.1916662)
Supplement: Supplementary file 1 [file Supplementary_File_1.docx]

**Supplemental materials**

**The characterization of zein complex system loaded with *Venenum Bufonis***

Peizhi Mao ^1†^, Shiyin Chen ^2†^, Fuge Niu ^2^, Zhaie Lu^1*^, Weichun Pan ^2*^

^1^Women and Children’s Hospital of Ningbo University, Ningbo , China , 315012

^2^ College of Food Science and Biotechnology, Zhejiang Gongshang University, Hangzhou, China, 310018

*** Correspondence:**
Zhaie Lu*
[feyyluzhaie@nbu.edu.cn](mailto:feyyluzhaie@nbu.edu.cn)

Weichun Pan*
Panweichun1970@163.com

†These authors contributed equally to this work and share first authorship

**XRD analyses**

XRD patterns of zein-*VB* MCS were prepared using an X-ray diffractometer (Rigaku MiniFlex 600C, Rigaku Holdings Corporation, Tokyo, Japan) equipped with Cu Kα radiation at a wavelength of 1.5406 Å. Measurements were obtained at 40 kV, 30 mA, room temperature with a scanning rate of 0.02°/s and a diffraction angle range of 5 to 90° (2θ range), where θ is the angle of incidence of the X-ray beam on the sample.

Figure S1 SEM images of Zein-*VB*_5:1_.

Figure S2 SEM images Zein-*VB*_10:1_.

Figure S3 SEM images of Zein-*VB*_20:1_.

Figure S4 SEM images of Zein-*VB*_30:1_.

Figure S5 X-ray diffraction patterns of 4 samples. The various colors and geometrical shapes stand for different zein-*VB* MCS. Black and square stands for Zein-*VB*_50:1_, red and circle for Zein-*VB*_10:1_, green and triangle for Zein-*VB*_20:1_, and blue and diamond for Zein-*VB*_30:1_.

Figure S6 Fitting operations upon Fig. 1e, (a) The first order; (b) Higuchi. Black and square stands for Zein-*VB*_30:1_, red and circle for Zein-*VB*_20:1_, pink and triangle for Zein-*VB*_5:1_, and blue and diamond for Zein-*VB*_10:1_.
